# Comparison of the diagnostic accuracy of HE4 with CA125 and validation of the ROMA index in differentiating malignant and benign epithelial ovarian tumours among patients in Lagos, Nigeria

**DOI:** 10.3332/ecancer.2023.1568

**Published:** 2023-07-05

**Authors:** Khadijah Adebisi Shittu, Kabiru Afolarin Rabiu, Oluwarotimi Ireti Akinola, Saheed Bolaji Ahmed, Adeniyi Abiodun Adewunmi

**Affiliations:** 1 Department of Obstetrics and Gynaecology, Lagos State University Teaching Hospital, Ikeja 100271, Lagos State, Nigeria; 2 Department of Obstetrics and Gynaecology, Lagos State University College of Medicine, Ikeja 100271, Lagos State, Nigeria; 3 Department of Community Medicine, University College Hospital, Ibadan 200212, Oyo State, Nigeria

**Keywords:** human epididymis protein 4, cancer antigen 125, risk of malignancy algorithm, ovarian cancer

## Abstract

This prospective cross-sectional study compared the diagnostic accuracy of human epididymal protein 4 (HE4) with cancer antigen 125 (CA 125) and validates the risk of malignancy algorithm (ROMA) in differentiating benign from malignant ovarian tumours. The study population included 112 women with an ultrasound diagnosis of an adnexal mass, out of whom 49 women had a diagnosis of ovarian cancer following optimal debulking surgery, and 63 women had a diagnosis of benign ovarian tumour. All diagnosis was confirmed by histopathological analysis. Serum HE4 and CA 125 were assessed preoperatively according to the manufacturer’s instructions. CA 125 and HE4 cut-offs were 35 U/mL and 70 pM/L respectively. Serum CA 125 and HE4 were significantly higher in ovarian cancer patients compared to those with benign ovarian tumours (*p* < 0.001 and *p* < 0.000, respectively). HE4 had higher sensitivity (77.5% versus 69.4%), specificity (96.8% versus 82.5%), positive predictive value (PPV) (95% versus 75.6%) and negative predictive value (84.7% versus 77.6%) than CA 125. When the two markers were combined with each other in the ROMA index, Specificity and PPV reached 100% each. In the receiver operative characteristics analysis, the area under the curve for CA 125 was 0.679 (95% CI 0.566–0.791, *p *= 0.001), HE4 was 0.845 (95% CI 0.760–0.930, *p *= 0.000) and ROMA was 0.902 (95% CI 0.851–0.998, *p *= 0.000) and this was statistically significant (*p *< 0.001). Conclusively, HE4 performed better than CA 125 in differentiating benign from malignant ovarian tumours and the combination of the two biomarkers improved the detection of ovarian cancer. In addition, the cut off values corresponding to the highest accuracy for CA 125 and HE4 were 126 U/mL and 42 pM/L respectively in this study. The value for CA 125 is much higher while that of HE4 is much lower than the reference values obtained predominantly from the white population.

## Introduction

Ovarian cancer is the 8th most common cancer among women and accounts for 3.4% of all cancers in women in 2020 [1]. It is the third most common cancer of the female genital tract yet remains the leading cause of gynaecological malignancy-related mortality worldwide [1]. The incidence has been rising gradually with incidence expected to double by 2024 [2].

Diagnosis of ovarian cancer still poses a great challenge to clinicians because of the intra-abdominal disadvantaged position of the ovary. Also, symptoms are vague in the early stage: many patients present with gastrointestinal symptoms which causes a lot of misdiagnoses [3]. Consequently, most ovarian cancers remain clinically undetected until patients have developed late-stage disease and only a mere 25% of cancers are detected as stage I disease [4]. The development of an ovarian cancer-specific tumour marker for the early detection of the disease has the capacity to improve the survival rate.

The ovarian biomarker cancer antigen 125 (CA 125) has been extensively studied regarding ovarian cancer screening, detection and progression. First described in 1981 by Bast *et al* [5] by developing a monoclonal antibody against the antigen, a radioimmunoassay was developed in 1983 to detect serum levels using a threshold of 35 u/mL [6]. However, CA 125 as a biomarker has some limitations. It is not elevated in about half of early-stage ovarian cancers and is elevated in many benign gynaecological diseases such as benign ovarian tumours, pelvic inflammatory disease, endometriosis and even physiological conditions like pregnancy and menstruation. It may also be elevated in some medical conditions [7, 8]. Consequently, there have been reports that the sensitivity, specificity, and positive predictive values (PPV) of CA 125 are not very impressive. Moreover, these indices are much better in postmenopausal women than premenopausal women [8]. New biomarkers could therefore contribute to the accuracy of CA 125 and be useful as an adjunct to CA 125.

Human epididymis protein 4 (HE4) is a glycoprotein encoded by the whey acidic four-disulfide core domain protein 2 (WFDC2) gene. Studies demonstrated the presence of HE4 in the female genitourinary tract, the respiratory tract, the renal epithelium, and the salivary ducts. Despite its wide distribution, it is over-expressed only in pathological tissue, and it has been reported to demonstrate good sensitivity and specificity in detecting ovarian cancer [9].

The risk of ovarian malignancy algorithm (ROMA) takes into consideration the concentration of the two biomarkers and the patient's menopausal status to generate a score, which translates to a high or low likelihood of finding a malignancy based on established cutoffs [10]. Women with ROMA scores above the cutoff have an increased risk of ovarian cancer and should be referred to a gynaecological oncologist prior to surgery [10].

Previous studies done on the role of HE4 in discriminating benign from malignant ovarian tumours have reported conflicting reports [11–14]. Furthermore, these studies were notably done in the white population. Also, we do not know what constitutes normal and abnormal levels of HE4 in blacks. Hence, there is a need for more studies on the subject matter especially in the black population.

This study therefore aims to compare the diagnostic characteristics of HE4 and CA 125 in malignant and benign epithelial ovarian tumours, and to validate the ROMA index in discriminating epithelial ovarian cancer from benign ovarian tumours in Lagos, Nigeria.

## Methods

### Study design

This was a prospective cross-sectional study.

### Study population

The study included 112 patients who presented with ovarian masses at the Lagos State University Teaching Hospital between March 1, 2020, and December 31, 2020. All patients underwent imaging by pelvic ultrasound scan to document the presence of an ovarian mass. Women with a previous history of bilateral oophorectomy, chemotherapy, radiotherapy, or hormonal therapy for other malignancies were excluded from the study.

### Serum samples and marker assays

Phlebotomy was done before the commencement of any medications. 10 mL of venous blood samples were obtained from all the patients following an overnight fast before surgery. Thereafter, centrifugation was done at 2,500 rpm for 10 minutes. The serum was then withdrawn and frozen at −20°C until analysis. Serum samples collected from all the patients who met the inclusion criteria were analysed for both CA 125 and HE4.

CA 125 analysis was done on The Abbott Axsym system (Abbott Diagnostics Division, Chicago) based on Micro Particle Enzyme Immunoassay technology.

HE4 assay was done on the fully automated ARCHITECT instrument (Abbott Diagnostics Division, Chicago) based on Electro-Chemi Luminescent micro particle Immunoassay.

Cut-off values was set according to the indications of the manufacturer at 35 IU/L for CA 125 while for HE4 at ≤70  pmol/L.

ROMA is a predictive probability algorithm that classifies women with ovarian mass as being at high or low risk of ovarian cancer based on the combination of serum values of CA 125, HE4 and menopausal status.

ROMA classifies patients as being at a low or at a high risk for malignant disease using the following algorithms:

Premenopausal: predictive index (PI) = −12.0 + (2.38 × LN(HE4)) + (0.0626 × LN(CA 125))

Postmenopausal: PI = −8.09 + (1.04 × LN(HE4)) + (0.732 × LN(CA 125))

Predicted probability: (PP) = 100 × exp (PI)/(1 + exp (PI))

HE4 and CA 125 values were input into the ROMA calculator software with automatic calculation of the corresponding ROMA index [15]. According to the manufacturer’s insert, the following thresholds were selected for ROMA predictive probability:

Pre-menopausal women:

* PP ≥11.4% = high risk of finding ovarian cancer

* PP <11.4% = low risk of finding ovarian cancer

Post-menopausal women:

* PP ≥29.9% = high risk of finding ovarian cancer

* PP <29.9% = low risk of finding ovarian cancer

### Patients

The patients recruited had surgical removal of the ovarian mass and diagnosis was confirmed from histological report of the removed specimen. Patients with suspected ovarian malignancy had staging laparotomy done. The surgeries were done by the gynaecology oncology team led by a consultant gynaecologist. The surgical procedures in benign cases included resection of the cyst or unilateral/bilateral oophorectomy, and in the malignant cases total abdominal hysterectomy, bilateral salpingo-oophorectomy, infracolic omentectomy and pelvic and para-aortic lymphadenectomy when indicated. Cytologic analysis of ascitic fluid or when absent of peritoneal washing was performed. Optimal debulking is defined as residual tumour less than or equal to 1 cm in maximum diameter. Tumour was staged according to 2009 International federation of gynaecology and obstetrics staging system and histologically defined according to WHO classification.

### Data processing and analysis

Data was collated daily, checked for completeness and all errors corrected immediately. The data was entered and analysed using statistical package for social sciences version 22. Descriptive statistics, tables, charts and graphs were used to summarise variables. Uniformly distributed data were summarized using the mean and SD, while data that are not uniformly distributed were summarised using median and inter quartile range. Continuous variables were compared using the student *T* test. Categorical variables were compared using the chi square test. The cut off point for CA 125, HE4 and ROMA that will probably distinguish between benign and malignant ovarian tumours was determined using the area under the receiver operator characteristic (ROC). Sensitivity, specificity, PPV, negative predictive values (NPV) were also calculated. The level of statistical significance was set at 5%.

### Ethical considerations

An ethical approval was obtained from the Health Research Ethics Committee of the Lagos State University Teaching Hospital before the recruitment of participants.

## Results

Table 1 shows the socio-demographic and gynaecological characteristics of the study participants. Approximately 43% of the study participants were aged 40–59 years while only 15.4% of them were aged <20 years. The mean age was 42.54 ± 13.75 years. Fifty (44.6%) of them had skilled occupation while 45.5% had tertiary education. Of all the study participants, 14(12.5%) had a positive family history of breast cancer, while 11(9.8%) had a family history of ovarian cancer. Seventy-three (65.2%) of the participants were premenopausal while 39(34.8%) were post-menopausal. The modal parity group was 1–4 with a percentage of 42%.

The serum levels of HE4, CA 125 and calculated ROMA index were all significantly higher in the epithelial ovarian cancer group than those in the benign ovarian disease group and this was statistically significant (*p*-value = 0.000, 0.001 and 0.000, respectively). This is depicted in Table 2.

Table 3 shows the sensitivity, specificity, positive and NPV of CA 125, HE4 and ROMA index. HE4 had higher sensitivity and specificity than CA 125 (77.5% versus 69.4% and 96.8% versus 82.5%, respectively). Also, the PPV and NPV of HE4 were higher than CA 125 (95% versus 75.6% and 84.7% versus 77.6%, respectively). Specificity and PPV were increased to 100% each when the two markers were combined with each other in the ROMA index.

**Figure 1.**
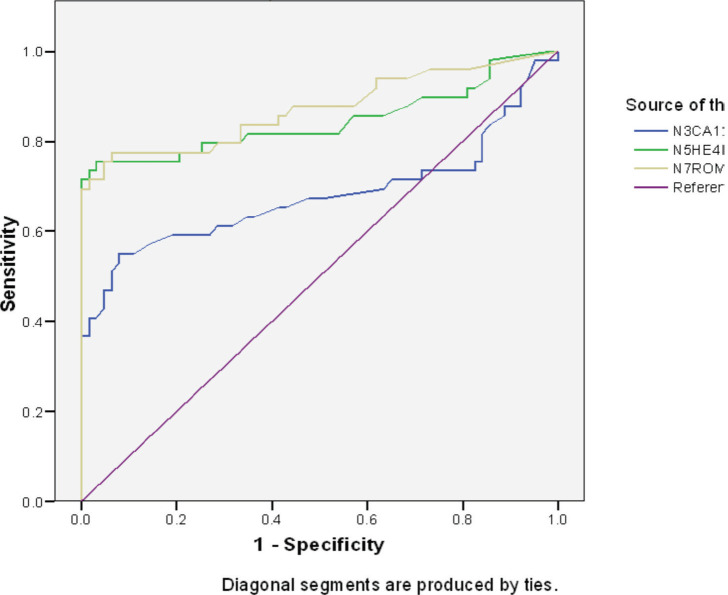
ROC curve of CA125, HE4 and ROMA index.

Table 4 and Figure 1 depicts the area under the curve (AUC) for the ROC of CA 125, HE4 and ROMA index in the diagnosis of epithelial ovarian cancer. The AUC for HE4 was 0.845 (95% CI 0.760–0.930, *p* = 0.000). This shows that it is a good test for differentiation of malignant from benign epithelial ovarian tumours, while that of CA 125: 0.679 (95% CI 0.566–0.791, *p* = 0.001) shows that it is a fair test for differentiation of malignant from benign epithelial ovarian tumours. Additionally, the AUC for the combination of the two serum markers (ROMA) was 0.902 (95% CI 0.851–0.998,* p* = 0.000) which shows that it is an excellent test for differentiation of malignant from benign epithelial ovarian tumours.

Table 5 shows the cut off values for CA 125, HE4 and ROMA index obtained from this study, above which an epithelial ovarian tumour is likely to be malignant.

## Discussion

Ovarian cancer is an enigmatic disease characterised by late presentation and advanced disease. at presentation. Diagnosis of ovarian cancer still poses a great challenge to clinicians because of the intra-abdominal disadvantaged position of the ovary. Also, symptoms are vague and non-specific in the early stage of the disease, and it is often difficult to make a diagnosis of benign or malignant ovarian tumour pre-operatively [16].

CA 125 has been used universally in the diagnosis of ovarian cancer while HE4 and ROMA index have been tested mainly in the white population. Considering that what constitute normal findings in white population may be different from what obtains in blacks, this prospective cross-sectional study was therefore carried out to compare the accuracy of HE4 with CA 125, and the risk stratification tool: ROMA, to differentiate benign from malignant epithelial ovarian tumours prior to surgical intervention and therefore aid appropriate referrals.

This study found a significant difference between benign and malignant epithelial ovarian tumours with respect to HE4 and ROMA levels, hence, the study suggests a possible role for the use of HE4 and ROMA index as a diagnostic marker for detecting epithelial ovarian cancer.

This study revealed that serum HE4 levels were higher in the epithelial ovarian cancer group as compared with the benign ovarian disease group and this finding was statistically significant. This finding is consistent with the findings of a previous study by Montagnana *et al* [17] in Verona, Italy. In this prospective observational study, it was reported that the mean serum level of HE4 was significantly higher than those in the benign ovarian tumour group. several other studies also reported similar findings [10, 12, 13, 18].

In this study, HE4 had a higher sensitivity, specificity, PPV, and NPV when compared with CA 125. This finding agrees with that of Hamed *et al* [19] in Egypt who reported a similar higher sensitivity (90% versus 83.3%), specificity (95% versus 85%), PPV (93.1% versus 80.7%) and NPV (92.7% versus 87.2%) of HE4 when compared to CA 125. However, Zhang *et al* [20] in China and Pitta *et al* [21] in Brazil reported CA 125 to have a higher sensitivity, specificity, positive and NPV than HE4.

The limitation of the use of a single tumour marker in making a diagnosis of ovarian cancer has been addressed in several clinical scenarios, hence the need to switch to a multi-marker approach in clinical practice to achieve better diagnostic accuracy. Thus, when both tumour markers were combined in the ROMA predictive probability, higher specificity and PPV were obtained. Anastasi *et al* [22] in their study also reported a specificity and PPV of 100% for ROMA. Higher specificity means that it is likely to predict the absence of ovarian cancer better than either tumour marker alone. Improvement in PPV has the resultant effect of reducing inappropriate referrals and its associated cost and waste of time. It also reduces the number of midline laparotomies which is still the standard of care in suspected ovarian cancer, thereby affording patients the use of more cosmesis-appealing incisions.

The diagnostic accuracy of CA 125, HE4 and by extension the ROMA index in differentiating benign from malignant ovarian tumours were verified using the ROC analysis. The resultant AUC values of CA 125, HE4 and ROMA show that CA 125 is a fair test, HE4 is a good test while ROMA is an excellent test for distinguishing malignant from benign ovarian tumours. Thus, ROMA performed better than either tumour marker alone. This means that HE4 and ROMA improved the diagnosis of ovarian cancer when compared to the current standard in this environment, CA 125. This is similar to the results of Moore *et al* [23] who found that a combination of CA 125 and HE4 performed better than CA 125 alone. However, a study done by Gorp *et al* [24] in Belgium reported that neither HE4 nor ROMA performed better than CA 125 in detecting ovarian cancer. It is therefore highly desirable that ROMA be used to evaluate ovarian cancer pre-operatively, but in our environment which is a developing country with limited resources, even though ROMA is desirable, where patients cannot afford the two tests, HE4 alone may be used in place of CA 125 because it has a better diagnostic accuracy.

This study also aimed to find the cut off points for the tumour markers. The cut off values corresponding to the highest accuracy for CA 125, HE4 and ROMA were 126 U/mL, 42 pM/L and 16.7% respectively, although these values are different from what obtained in other studies [11, 23], they were established at the point on the ROC curve where the least false negative and false positive results existed. Probably, the difference is because of inherent racial differences in the population studied. Notably, significantly higher than the cut-off used in clinical practice is the cut-off value of CA 125 obtained from this study. This may be one of the reasons why there is a lot of misdiagnoses of ovarian cancer in our environment as CA 125 is the major tumour marker used in diagnosing epithelial ovarian cancer, and the reference value was obtained following studies done mainly in the white population [7, 13, 21, 25]. A lot of patients with CA 125 greater than 35 U/mL end up not having malignant disease after histological examination. However, it is difficult to generalise this finding amongst the Nigerian, and at large, the black population mainly because of the small sample size, and the uni-centre nature of this study, hence there may be needed for larger scale studies mainly in the black population to determine what constitute the cut off point for CA 125 in the diagnosis of ovarian cancer.

This study has some limitations, it is an institution-based study with a relatively small sample size which may not be representative of the general population, thus a larger scale multicentred study is required to further validate the findings of this study.

## Conclusion

HE4 demonstrated better diagnostic accuracy than CA 125 as a tumour marker for differentiating benign from malignant ovarian tumours. It demonstrated better sensitivity, specificity, positive and NPV. HE4 improves the utility of CA 125 and the combination of the two biomarkers in the ROMA index further improved the diagnostic accuracy. The use of this combination may help in the discrimination of benign from malignant ovarian tumours as compared with the use of either tumour marker alone, thus facilitating appropriate referrals for definitive care. In addition, the cut off values corresponding to the highest accuracy for CA 125 and HE4 were 126 U/mL and 42 pM/L respectively in this study. The value for CA 125 is much higher while that of HE4 is much lower than the reference values obtained predominantly from the white population, probably due to inherent racial differences.

## Conflicts of interest

The authors declare that they have no conflicts of interest.

## Declaration of funding

None.

## Figures and Tables

**Table 1.** Distribution of socio-demographic and gynaecologic characteristics of the study participants.

Characteristic	Frequency	Percentage
Age (in years) <20 20–39 40–59 60 and above Mean = 42.54 ± 13.75 Range = 10–71 years	6 42 48 16	5.4 37.5 42.9 14.2
Religion Christianity Islam	57 55	50.9 49.1
Tribe Hausa Ibo Yoruba Others	11 32 44 25	9.8 28.6 39.3 22.3
Occupation Skilled Semiskilled Unskilled	50 42 20	44.6 37.5 17.9
Education Primary Secondary Tertiary	20 41 51	17.9 36.6 45.5
Family history Breast cancer Ovarian cancer Colorectal cancer	14 11 5	12.5 9.8 4.5
Menopausal status Premenopausal Post menopausal	73 39	65.2 34.8
Parity 0 1–4 5 and above	46 47 19	41.0 42.0 17.0

**Table 2.** Serum levels of HE4, CA125 and ROMA index among patients with benign and malignant ovarian tumours.

Parameters	Benign ovarian tumour group (*n* = 63)	Ovarian cancer group (*n* = 49)	*p* value
HE4 median(IQR)	18(23)	95(108)	0.000
CA 125 median(IQR)	88(76)	198(392)	0.001
ROMA index median(IQR)	1(6)	46(47)	0.000

IQR = Inter quartile range

**Table 3.** Sensitivity, specificity, positive and NPV for CA125, HE4 and ROMA index.

	Sensitivity(%)	Specificity(%)	PPV(%)	NPV(%)
CA125	69.4	82.5	75.6	77.6
HE4	77.5	96.8	95.0	84.7
ROMA	75.5	100	100	84.0

**Table 4.** Comparison of AUC from the ROC curve analysis for serum CA125, HE4 and ROMA levels.

Characteristics	ROC-AUC	Lower limit	Upper limit	*p* value
HE4	0.845	0.760	0.930	0.000
CA125	0.679	0.566	0.791	0.001
ROMA	0.902	0.851	0.998	0.000

**Table 5.** Cut off points for the analytes for making a diagnosis of ovarian cancer.

Tumour marker	Cut off
CA125	126 U/ML
HE4	46.5P M/L
ROMA	16.7%
